# An Ultra-Wideband THz/IR Metamaterial Absorber Based on Doped Silicon

**DOI:** 10.3390/ma11122590

**Published:** 2018-12-19

**Authors:** Huafeng Liu, Kai Luo, Shihao Tang, Danhua Peng, Fangjing Hu, Liangcheng Tu

**Affiliations:** 1 MOE Key Laboratory of Fundamental Physical Quantities Measurement, Huazhong University of Science and Technology, Wuhan 430074, China; huafengliu@hust.edu.cn (H.L.); tangshihao@hust.edu.cn (S.T.); flower0614@163.com (D.P.); tlc@hust.edu.cn (L.T.); 2 Hubei Key Laboratory of Gravitation and Quantum Physics, School of Physics, Huazhong University of Science and Technology, Wuhan 430074, China; 3 School of Electronic Information and Communications, Huazhong University of Science and Technology, Wuhan 430074, China; kluo@hust.edu.cn

**Keywords:** terahertz, ultra-wideband, absorber

## Abstract

Metamaterial-based absorbers have been extensively investigated in the terahertz (THz) range with ever increasing performances. In this paper, we propose an all-dielectric THz absorber based on doped silicon. The unit cell consists of a silicon cross resonator with an internal cross-shaped air cavity. Numerical results suggest that the proposed absorber can operate from THz to far-infrared regimes, having an average power absorption of ∼95% between 0.6 and 10 THz. Experimental results using THz time-domain spectroscopy show a good agreement with simulations. The underlying mechanisms for broadband absorption are attributed to the combined effects of multiple cavities modes formed by silicon resonators and bulk absorption in the doped silicon substrate, as confirmed by simulated field patterns and calculated diffraction efficiency. This ultra-wideband absorption is polarization insensitive and can operate across a wide range of the incident angle. The proposed absorber can be readily integrated into silicon-based photonic platforms and used for sensing, imaging, energy harvesting and wireless communications applications in the THz/IR range.

## 1. Introduction

Terahertz (THz) absorbers with broadband operations are essential components for various applications such as sensing, imaging, energy harvesting and wireless communications. Although some materials in nature have shown reasonably good absorptions within the THz regime, it is still in great demand of ultra-wideband absorbers with flat and high power absorptance. With the development of metamaterials [1], different material properties and functionalities have been demonstrated [2,3,4,5]. Metamaterial-based perfect absorbers (MPAs) have been extensively studied since it was first proposed in the microwave range by Landy et al. [6]. Over the past years, the performances of MPAs have been increased and the operating frequencies have been expanded from microwaves to THz, infrared and visible ranges due to excellent scalability of metamaterials [7,8,9,10,11].

The unit cell of conventional MPAs usually consists of a metallic resonator, a dielectric spacer and a ground layer. At its resonant frequency, near-unity power absorptance can be achieved as both the power transmittance and reflectance are minimised. However, MPAs with metallic resonators normally exhibit narrow bandwidths because of their resonating nature. For broadband operation, either complicated unit cell shapes, or composite and multilayer structures are required, limiting the applications for the increased fabrication and design complexities. To tackle this problem, doped silicon substrates have been used to create broadband MPAs. Different unit cells patterns, including circular holes [12], rectangular cubes [13,14,15], cross-cave patches [16] sawtooth structures [17], crosses [18,19] and dumbbell shapes [20], have been demonstrated. The broadband absorptions are mainly due to the excitation of plasmonic waveguide modes or by multi-interference and diffraction effects [21]. The electromagnetic (EM) responses of the devices can be further engineered by changing the parameters of the unit cell or by adjusting the doping concentration.

Although silicon-based absorbers have been extensively investigated, most of the work mainly focused on the EM responses only within the THz region, i.e., from 0.3 to 3 THz. Their absorption characteristics were not fully revealed in the far-infrared spectrum. With a wider operating spectrum, applications such as bolometric imaging, stealth applications and energy harvesting can be fully explored. Therefore, it is still in great demand for designing ultra-wideband absorbers within the THz/IR spectral ranges.

In this paper, we propose an ultra-wideband absorber based on a standard 400-μm thick doped silicon substrate and demonstrate its EM responses in the THz and far-infrared spectra (e.g., 0.1–10 THz). The unit cell consists of a silicon cross structure with an internal cross-shaped air cavity, creating multiple air-cavity modes to reduce the reflection over a large bandwidth. Numerical results suggest an average power absorptance of ∼95% from 0.6 to 10 THz, and a consistent performance across a wide range of the incident angle for both TE and TM polarisations can be obtained. Terahertz time-domain spectroscopy (THz-TDS) measurements from 0.2 to 2.5 THz show a good agreement with simulated results. When compared with previous studies, the proposed design has advantages such as single-layer resonating structure, low fabrication complexity and ultra-wide bandwidth, and can be readily integrated into silicon-based photonic platforms.

## 2. Design and Simulations

A 400-μm thick doped silicon substrate with a resistivity of 0.01–0.02 Ω·cm is used to fabricate the absorber. The complex dielectric constant of silicon can be described by Drude model as

$$\epsilon =\epsilon_{\infty }-\frac{{\omega_{p}}^{2}}{\omega^{2}+j\omega \gamma }$$

where $\epsilon_{\infty }$ = 11.68 is the permittivity of silicon at high frequencies, $\omega_{p}$ = 2π × 7.88 THz is the plasma frequency, and γ=2π×1.78 THz is the collision frequency [19].

**Figure 1:**
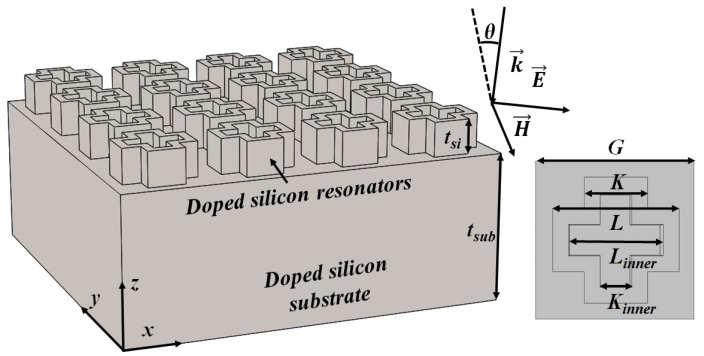
Schematic drawing of the proposed THz absorber.

As shown in Figure 1, the unit cell of the proposed absorber consists of a silicon cross resonator with an internal cross-shaped air cavity. Initial geometry parameters can be obtained by determining the lattice constant *G* and resonant frequencies of the cavities. Then, full-wave simulations were performed using CST Microwave Studio (Computer Simulation Technology GmbH, Darmstadt, Germany), where the unit cell boundary condition was applied to mimic an infinite two-dimensional array. In the simulations, the incident wave is TE-polarised (x-direction) and the propagation direction is along z-direction, where θ is the incident angle.

To accelerate the design procedure, we further applied a genetic algorithm [22] interfacing with CST to obtain the final design parameters. Normal incidence was used in the optimization procedure. The fitness function was to minimize the average power reflectance over the 0.6 to 10 THz region, which can be expressed as

$$fitness=\min(\sum_{\omega }|S_{11}\left(\omega \right){|}^{2}){|}_{0.6\;\mathrm{THz}<\omega <10\;\mathrm{THz}}$$

Subsequently, the power responses, including the power transmittance $T\left(\omega \right)=|S_{21}{\left(\omega \right)|}^{2}$, power reflectance $R\left(\omega \right)=|S_{11}{\left(\omega \right)|}^{2}$ and power absorptance A(ω)=1−T(ω)−R(ω), can be obtained from simulated S-parameters. Here, optimized parameters are *G* = 210 μm, *L* = 160 μm, and *K* = 80 μm. The length and width of the inner cavities are $L_{inner}$ = 120 μm, and $K_{inner}$ = 40 μm, while the thicknesses of the crosses and the substrate are $t_{Si}$ = 75 μm and $t_{Sub}$ = 325 μm, respectively.

**Figure 2:**
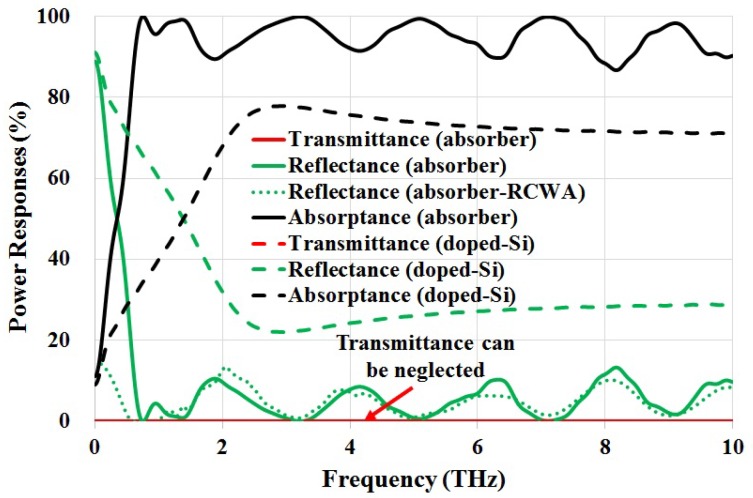
Simulated power responses for the proposed absorber and a bare doped silicon substrate.

Figure 2 shows the power responses of the proposed THz absorber, as well as a unpatterned silicon substrate of the same 400 μm thickness. It is seen that the power absorptance for an unpatterned silicon wafer improves as the frequency increases, reaching to its maximum of ∼78% at 2.8 THz, and then starts decreasing to about 70%. For the proposed absorber, the power absorptance becomes greater than 90% from 0.6 THz, and sustains a high value across the entire spectrum of interest up to 10 THz. The calculated average absorptance within 0.6–10 THz is ∼95%. Within this region, the power transmittances for both the proposed absorber and bare silicon ($t_{Sub}$ = 325 μm) can be neglected.

**Figure 3:**
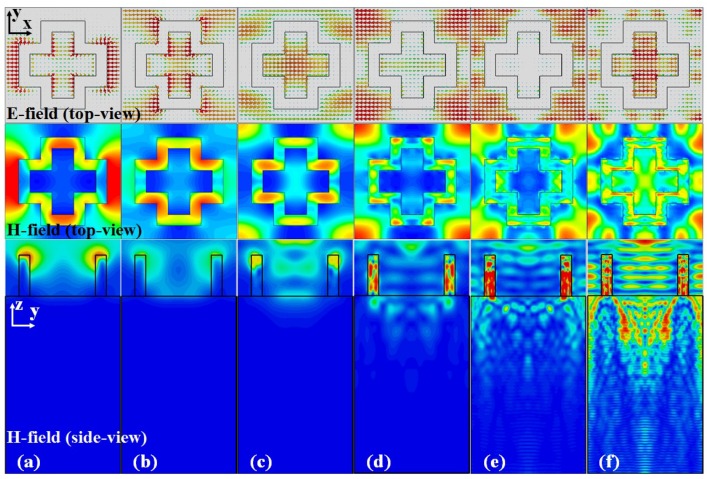
Simulated electric and magnetic field patterns for the absorber at (**a**) 0.75 THz, (**b**) 1.38 THz, (**c**) 3.23 THz, (**d**) 5.10 THz, (**e**) 7.13 THz and (**f**) 9.12 THz, respectively. The first and second row show the electric and magnetic fields (top-view), respectively, while the third row illustrates the magnetic fields (side-view).

In order to investigate the origins of these absorption peaks, instantaneous electric fields and average magnetic fields at the resonant frequencies are shown in Figure 3. The first and second row illustrate the top view of the electric and magnetic fields, respectively, and the last row is the side view of magnetic fields across the centre of the unit cell. It is clearly demonstrated that distinct field patterns were obtained at different resonant frequencies. At low frequencies, the absorption peaks were originated by different cavity modes. For example, the electric fields at the first resonance of 0.75 THz were localized between the left and right arms of the adjacent crosses, which can be treated as a parallel-plate plasmonic waveguide mode [23]. Figure 3b shows that the incident wave resonates at the top and bottom parts of the cross structure at *f* = 1.38 THz, providing a low reflectance. This frequency is not far from *f* = 1.43 THz, where the diffraction maximum in the air side is expected for a grating constant of *G* = 210 μm. However, in this case, most of the power is absorbed within the resonating structure, and will not produce a large reflected power even when the diffraction maximum occurs. As the frequency increases, field patterns become more complicated. In general, more incident energy propagates through the top cross-shaped resonators and then absorbed by the doped silicon substrate, as evidenced in Figure 3d–f. This shows the effectiveness of applying doped silicon substrates to achieve a high absorption within a wider operating bandwidth.

**Figure 4:**
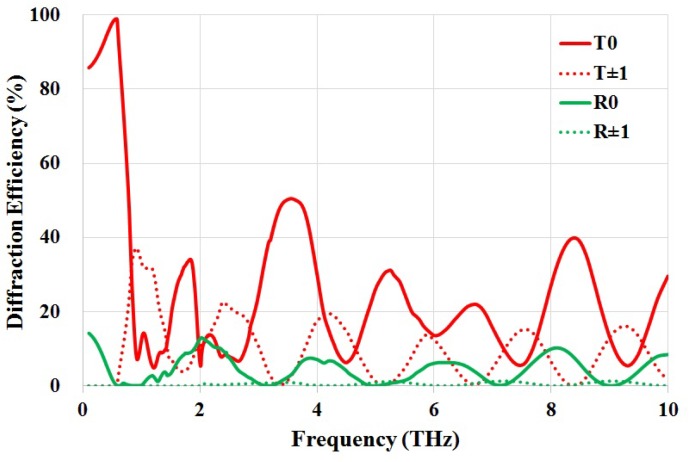
Calculated diffraction efficiency of the absorber using Rigorous Coupled Wave Analysis.

To further comprehend the absorption mechanisms of the proposed absorber, the diffraction effect is investigated using Rigorous Coupled Wave Analysis (RCWA). The absorber is treated as a two-dimensional grating and the diffraction efficiency of different diffraction orders (order 0 and ±1) are calculated, as shown in Figure 4. It is clearly seen that, in general, the diffraction maximum in reflection is small when compared to that in transmission, assuring a wideband absorption. Furthermore, the absorption peaks are in coincidence with the diffraction maximum in transmission, providing a minimum reflectance when the diffraction maximum in the substrate side occurs. Since the resistivity of the substrate is 0.01–0.02 Ω·cm, when the incident THz waves resonate with the high loss silicon grating, these resonance peaks are broadened.

The diffraction maximum can be estimated using G·sinθ=kλ/n, where *G* is the grating constant, θ is the diffraction angle, and *n* is the refractive index. Therefore, the frequency limit of diffraction in transmission and reflection can be calculated, which are $f_{T}$ = 0.42 THz and $f_{R}$ = 1.43 THz, respectively. This explains the rising edge of $T_{\pm 1}$ at *f*≈ 0.5 THz. It is also found that the contributions from $R_{\pm 1}$ are insignificant throughout the frequency of interest. Furthermore, at normal incidence, the absorption peaks over fixed frequency gaps are attributed to diffraction maximum T0 and $T_{\pm 1}$. This is caused by different grating constants originating from the silicon structure. As shown in Figure 2, the calculated total power reflectance (by considering diffraction order 0 and ±1) using RCWA agrees well with the simulated reflectance, further validating the modelling of this structure.

**Figure 5:**
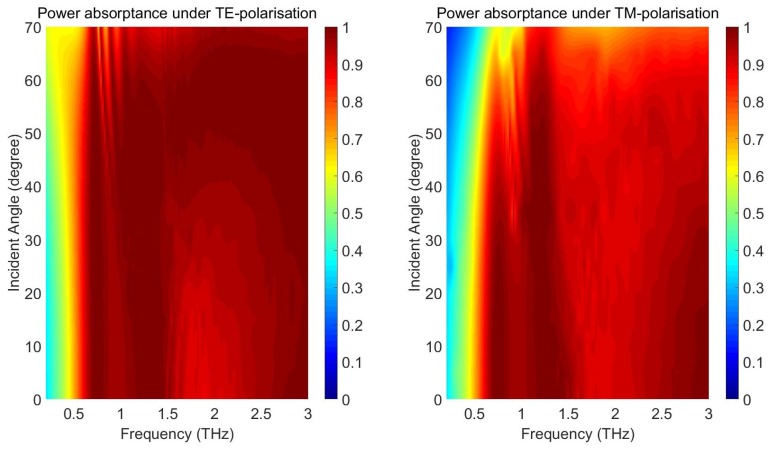
Simulated power absorptance as a function of frequency and incident angle.

As a four-fold symmetric structure, the proposed absorber is polarisation insensitive at normal incidence under both TE- and TM-polarisazitons. At oblique incidence, this structure is dependent on the polrisation as well as the incident angle due to the power imbalance within the cavity structures. Figure 5 shows the simulated power absorptance under different polarisations for the proposed THz absorber, by varying the frequency (0.2–3 THz) and incident angle ($0^{\circ }$–$70^{\circ }$). For TE-polarisation, the power absorption remains greater than 90% from 1 to 3 THz for incident angles up to $70^{\circ }$. Between 0.6 and 1 THz, the absorptance decreases to about 70% as the incident angle increases. The dependencies of the power absorptance spectra on the incident angle become more significant for TM-polarisation. From 1 to 3 THz, the absorptance remains greater than 90% for incident angles up to $50^{\circ }$ and then decreases to about 80% for a $65^{\circ }$ incidence. Nevertheless, the proposed absorber can operate over a large bandwidth for various incident angles.

## 3. Fabrication and Experimental Results

**Figure 6:**
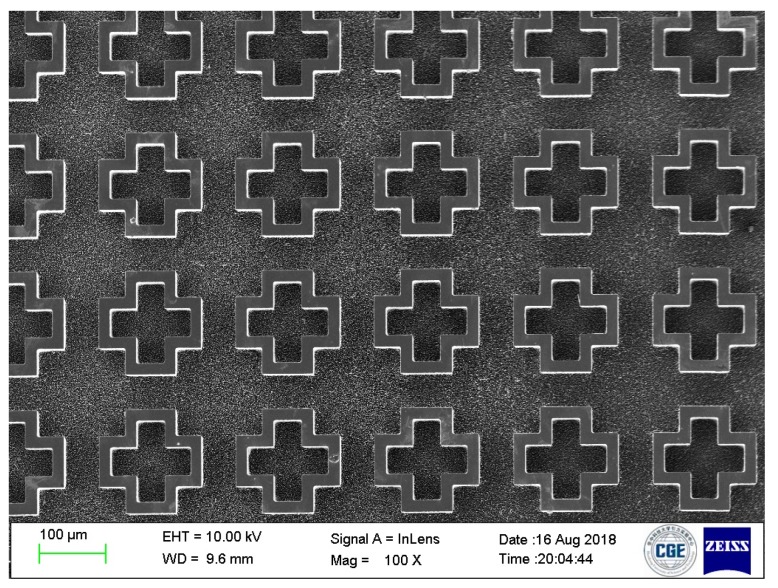
SEM image of the fabricated THz absorber.

The proposed design was fabricated using a 4-inch 400-μm thick silicon wafer. First, a 5-μm thick AZ9260 photoresist layer was spin-coated on the primer prepared wafer surface. Then, the wafer was exposed by ultraviolet (UV) light through the designed photomask. After the development process in the developer AZ400K, the exposed areas of the photoresist layer were striped, followed by a standard deep reactive ion etching (DRIE) process to etch silicon through the photoresist-free windows. Once the DRIE process is finished, the wafer was socked into acetone solution for at least 30 min to strip organic outgrowths and the residual photoresist. Finally, the 75-μm deep silicon trenches were formed on the substrate. The overall size of the fabricated sample chip was 19.95 mm × 19.95 mm with 95 × 95 (9025) unit cells. A scanning electron microscope (SEM) image of the sample is shown in Figure 6.

**Figure 7:**
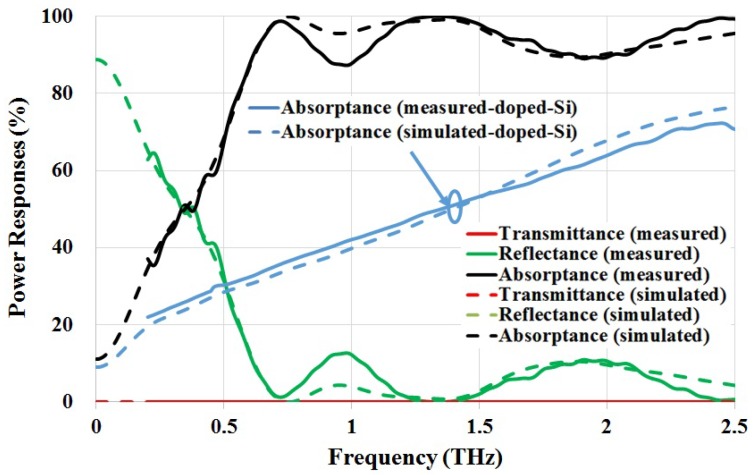
Measured power responses for the proposed THz absorber and doped silicon substrate.

Both transmission- and reflection-mode measurements of the proposed absorber and the doped silicon substrate were performed at normal incidence (TE-polarisation) using a Zomega Z-3 THz time-domain spectrometer (Zomega Terahertz Corporation, Troy, NY, US) with a spectral coverage from 0.2 to 2.5 THz. When compared with the bare doped silicon substrate, it is clearly shown that the proposed absorber can increase the absorptance due to multiple resonances and grating effects. As shown in Figure 7, measured results in general agree well with simulated values, having the designed resonant frequencies clearly identified. A slight redshift at the first resonance of 0.75 THz and a reduced power absorptance near 1 THz were observed. It is also seen that there is an excellent agreement between the simulated and measured absorptance of the absorber within the frequencies where the simulated and measured absorptance of doped silicon substrate agree well. Therefore, apart from the fabrication error, the discrepancies between simulated and measured results are also from the modelling of doped silicon dielectric properties in the simulation software.

In principle, this design can be operated within both the THz and infrared regions, as suggested by simulated results. This will be beneficial for various applications, including sensing [3], wireless communications [12], imaging [24], and energy harvesting [25], where wide bandwidth are essential. For example, in wireless communications systems, the reflected waves from the receiver side will reduce the signal-to-noise ratio (SNR) due to multi-path inteference [12]. The proposed design can be readily integrated into such systems and serve as an absorbing layer around the receiver to reduce such interference and thus improve the SNR performance.

It should be noted that at frequencies above 10 THz, simulated results show that the transmittance cannot be ignored anymore, and the absorptance will decrease. A possible way to obtain a high absorptance at the mid- and near-infrared regions is to fabricate vertically-alligned carbon nanotubes (VACNT) onto the silicon surface. The metallic VACNTs can serve as an absorptive material at high frequencies [26]. Furthermore, higher absorption can be achieved by employing additional capping layers for impedance matching between air and silicon layers [15]. Absorption peaks and operating frequencies can also be tuned by changing the doping concentration level of silicon [13].

## 4. Conclusions

In conclusion, we have designed, fabricated and experimentally demonstrated an ultra-wideband absorber within the THz and far-infrared spectra. The absorber was fabricated based on a standard 400-μm thick doped silicon substrate. Each unit cell contains a 75-μm thick silicon cross structure with an inner cross-shaped air cavity. The ultra-wideband absorption was achieved due to the combined effects of multiple cavity modes and bulk absorption of the doped silicon substrate. At low frequencies, different cavity modes were formed within this structure, significantly increasing the absorption when compared to unpatterned silicon substrate of the same thickness. At high frequencies, as more energy propagates through the top resonator, bulk absorption occurs within the silicon substrate due to its high doping concentration. This was confirmed by both the simulated field patterns and the calculated diffraction efficiencies using RCWA. The average power absorptance was simulated to be ∼95% from 0.6 to 10 THz, and agrees well with the THz-TDS measurements between 0.2 and 2.5 THz. This absorber is polarisation insensitive, and can sustain a high power absorptance for incident angles up to 50${}^{\circ }$ under both polarisations. More importantly, it can be readily integrated into other silicon-based photonic platforms for its low fabrication complexity, and can be used for sensing, wireless communications, imaging, and energy harvesting applications.

References

## References

[1] Zheludev N.I., Kivshar Y.S. (2012). From metamaterials to metadevices. Nat. Mater..

[2] Spada L.L., Vegni L. (2017). Near-zero-index wires. Opt. Express.

[3] Lee Y., Kim S.J., Park H., Lee B. (2017). Metamaterials and metasurfaces for sensor applications. Sensors.

[4] La Spada L., Vegni L. (2018). Electromagnetic Nanoparticles for Sensing and Medical Diagnostic Applications. Materials.

[5] Vakil A., Engheta N. (2011). Transformation Optics Using Graphene. Science.

[6] Landy N.I., Sajuyigbe S., Mock J.J., Smith D.R., Padilla W.J. (2008). Perfect metamaterial absorber. Phys. Rev. Lett..

[7] Watts C.M., Liu X., Padilla W.J. (2012). Metamaterial electromagnetic wave absorbers. Adv. Mater..

[8] Tao H., Landy N.I., Bingham C.M., Zhang X., Averitt R.D., Padilla W.J. (2008). A metamaterial absorber for the terahertz regime: Design, fabrication and characterization. Opt. Express.

[9] Avitzour Y., Urzhumov Y.A., Shvets G. (2009). Wide-angle infrared absorber based on a negative-index plasmonic metamaterial. Phys. Rev. B.

[10] Hao J., Wang J., Liu X., Padilla W.J., Zhou L., Qiu M. (2010). High performance optical absorber based on a plasmonic metamaterial. Appl. Phys. Lett..

[11] Aydin K., Ferry V.E., Briggs R.M., Atwater H.A. (2011). Broadband polarization-independent resonant light absorption using ultrathin plasmonic super absorbers. Nat. Commun..

[12] Kakimi R., Fujita M., Nagai M., Ashida M., Nagatsuma T. (2014). Capture of a terahertz wave in a photonic-crystal slab. Nat. Photonics.

[13] Pu M., Wang M., Hu C., Huang C., Zhao Z., Wang Y., Luo X. (2012). Engineering heavily doped silicon for broadband absorber in the terahertz regime. Opt. Express.

[14] Shi C., Zang X., Wang Y., Chen L., Cai B., Zhu Y. (2014). A polarization-independent broadband terahertz absorber. Appl. Phys. Lett..

[15] Yin S., Zhu J., Xu W., Jiang W., Yuan J., Yin G., Xie L., Ying Y., Ma Y. (2015). High-performance terahertz wave absorbers made of silicon-based metamaterials. Appl. Phys. Lett..

[16] Cheng Y., Huang M., Chen H., Guo Z., Mao X., Gong R. (2017). Ultrathin six-band polarization-insensitive perfect metamaterial absorber based on a cross-cave patch resonator for terahertz waves. Materials.

[17] Du L.H., Li J., Zhai Z.H., Meng K., Liu Q., Zhong S.C., Zhou P.W., Zhu L.G., Li Z.R., Peng Q.X. (2016). A high-performance broadband terahertz absorber based on sawtooth-shape doped-silicon. AIP Adv..

[18] Shi C., Zang X.F., Chen L., Peng Y., Cai B., Nash G.R., Zhu Y.M. (2016). Compact broadband terahertz perfect absorber aased on multi-interference and diffraction effects. IEEE Trans. Terahertz Sci. Technol..

[19] Cheng Y., Withayachumnankul W., Upadhyay A., Headland D., Nie Y., Gong R.Z., Bhaskaran M., Sriram S., Abbott D. (2015). Ultrabroadband plasmonic absorber for terahertz waves. Adv. Opt. Mater..

[20] Zang X., Shi C., Chen L., Cai B., Zhu Y., Zhuang S. (2015). Ultra-broadband terahertz absorption by exciting the orthogonal diffraction in dumbbell-shaped gratings. Sci. Rep..

[21] Cheng Y., Zou H., Yang J., Mao X., Gong R. (2018). Dual and broadband terahertz metamaterial absorber based on a compact resonator structure. Opt. Mater. Express.

[22] Chen P.Y., Chen C.H., Wang H., Tsai J.H., Ni W.X. (2008). Synthesis design of artificial magnetic metamaterials using a genetic algorithm. Opt. Express.

[23] Maier S.A. (2007). Plasmonics: Fundamentals and Applications.

[24] Fan K., Suen J.Y., Liu X., Padilla W.J. (2017). All-dielectric metasurface absorbers for uncooled terahertz imaging. Optica.

[25] Atwater H.A., Polman A. (2010). Plasmonics for improved photovoltaic devices. Nat. Mater..

[26] Ye H., Wang X.J., Lin W., Wong C.P., Zhang Z.M. (2012). Infrared absorption coefficients of vertically aligned carbon nanotube films. Appl. Phys. Lett..

